# microRNAs Make the Call in Cancer Personalized Medicine

**DOI:** 10.3389/fcell.2017.00086

**Published:** 2017-09-22

**Authors:** Simone Detassis, Margherita Grasso, Valerio Del Vescovo, Michela A. Denti

**Affiliations:** Laboratory of RNA Biology and Biotechnology, Centre for Integrative Biology, University of Trento Trento, Italy

**Keywords:** microRNAs, personalized medicine, cancer, MiR-SNP, biomarker

## Abstract

Since their discovery and the advent of RNA interference, microRNAs have drawn enormous attention because of their ubiquitous involvement in cellular pathways from life to death, from metabolism to communication. It is also widely accepted that they possess an undeniable role in cancer both as tumor suppressors and tumor promoters modulating cell proliferation and migration, epithelial-mesenchymal transition and tumor cell invasion and metastasis. Moreover, microRNAs can even affect the tumor surrounding environment influencing angiogenesis and immune system activation and recruitment. The tight association of microRNAs with several cancer-related processes makes them undoubtedly connected to the effect of specific cancer drugs inducing either resistance or sensitization. In this context, personalized medicine through microRNAs arose recently with the discovery of single nucleotide polymorphisms in the target binding sites, in the sequence of the microRNA itself or in microRNA biogenesis related genes, increasing risk, susceptibility and progression of multiple types of cancer in different sets of the population. The depicted scenario implies that the overall variation displayed by these small non-coding RNAs have an impact on patient-specific pharmacokinetics and pharmacodynamics of cancer drugs, pushing on a rising need of personalized treatment. Indeed, microRNAs from either tissues or liquid biopsies are also extensively studied as valuable biomarkers for disease early recognition, progression and prognosis. Despite microRNAs being intensively studied in recent years, a comprehensive review describing these topics all in one is missing. Here we report an up-to-date and critical summary of microRNAs as tools for better understanding personalized cancer biogenesis, evolution, diagnosis and treatment.

## MicroRNA biogenesis

microRNAs are small non-coding RNAs described for the first time in 1993 (Lee et al., 1993). They are found in plants (Jones-Rhoades et al., 2006), animals and viruses (Grundhoff and Sullivan, 2011), with functions in RNA silencing and post-transcriptional regulation of gene expression. They also have a role in pathological processes including neurodegenerative diseases (Molasy et al., 2016; Reddy et al., 2016) and cancer (da Silva Oliveira et al., 2016; Mohammadi et al., 2016). microRNAs transcriptional units are present both in introns or exons of other genes and as independent ones (Godnic et al., 2013). They are transcribed mainly by RNA Polymerase II, capped and polyadenylated forming primary microRNAs (pri-microRNAs). A small group is generated by RNA Polymerase III. The pri-microRNA is processed in a precursor microRNA (pre-microRNA)—about 70 nt—by RNase III Drosha and RNA-binding protein DGCR8 (Lee et al., 2003). Subsequently, the pre-microRNA is transported out of the nucleus via exportin-GTPase RAN system, where is further processed by Dicer producing the double-stranded microRNA of 22nt (Wilson et al., 2015). A complex made of AGO proteins is able to bind it and form the miRISC. Only one strand of the microRNA is loaded in the RISC complex, while the other (the passenger strand) is thought to be degraded. The RISC complex has an important post-transcriptional role in gene expression, regulating stability and turnover of mRNAs. The loaded microRNA can target mRNAs, exploiting its sequence complementarity. If the match is perfect the system leads to the mRNA degradation (Yekta et al., 2004), otherwise it impedes its translation (Ipsaro and Joshua-Tor, 2015). Because of their short length, microRNAs, which usually bind the 3′UTR of target mRNAs, are able to target several distinct mRNAs and, on the other hand, any given mRNA may present many binding sites for different microRNAs (Bartel et al., 2009).

## MicroRNA and cancer

**Figure 1:**
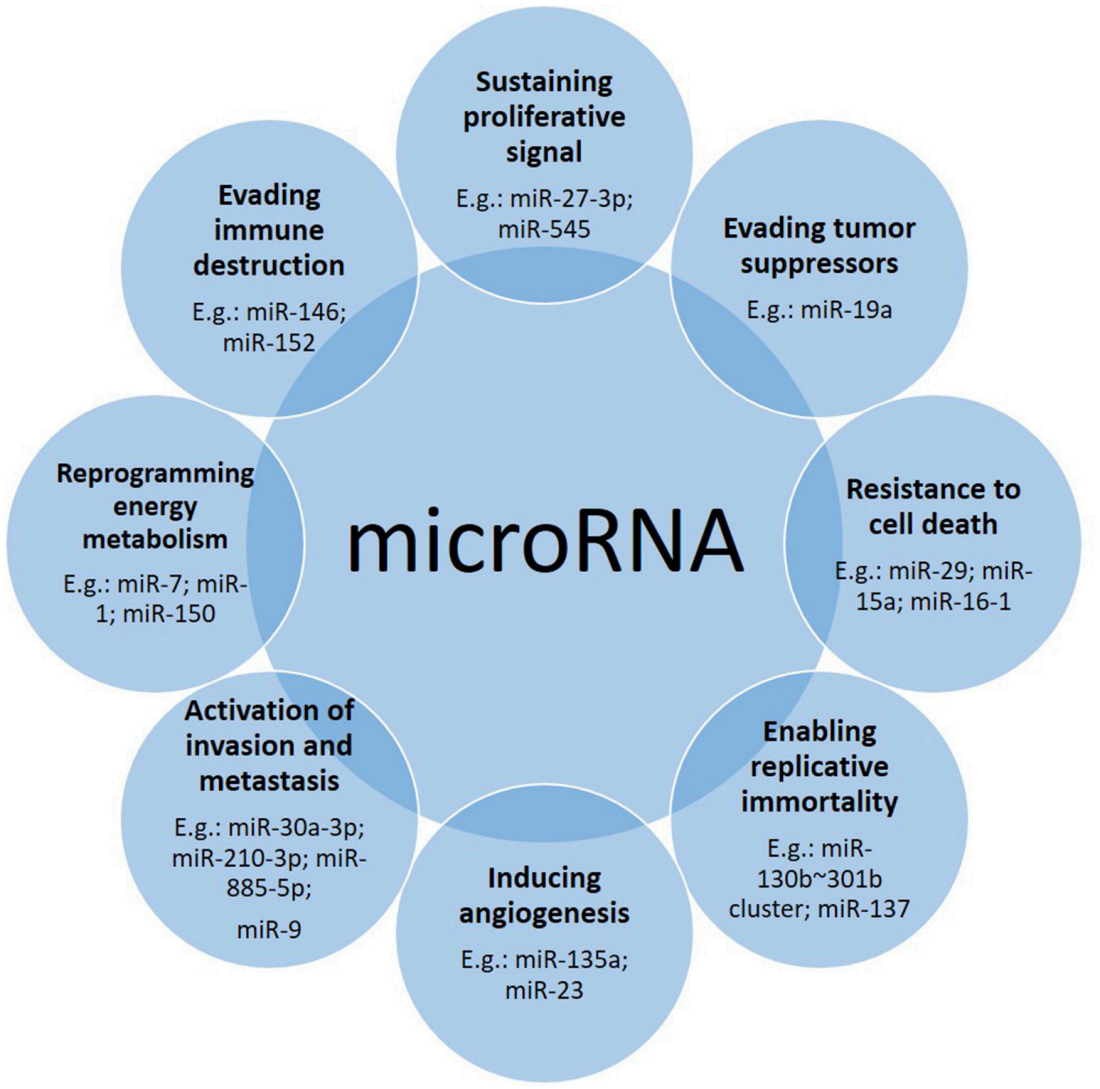
Down- or up-regulation of microRNAs contribute to the cancer driving steps. Often one microRNA affects more than one hallmark, with one prevailing tissue-dependent mechanism.

It has been widely reported that microRNAs are involved in many aspects related to cancer (Figure 1). Following the “hallmarks” of cancer (Hanahan and Weinberg, 2011) we can find many articles in which microRNAs play a role in each of these steps on the road of cancer biogenesis and progression. Here we describe some examples (Table 1).

1. *Sustaining the proliferative signal*. miR-27a-3p was shown to be associated with progression of nasopharyngeal cancer from patient samples and to be increased compared to healthy tissues. *In vitro* it promotes 5–8 F cell proliferation, migration and invasion targeting MAPK10 (Li and Luo, 2017). On the contrary, miR-545 was found decreased in colorectal cancer (CRC) in comparison to normal tissues and thus, its over-expression led to diminished proliferation and colony formation capacity (Huang and Lu, 2017). Luciferase and western blot assay confirmed the *in-silico* prediction of miR-545 targeting EGFR in CRC cell lines.
2. *Evading tumor suppressors*. Liu and colleagues (Liu Y. et al., 2017) showed how miR-19a binds directly the 3′UTR of TIA1 mRNA, involved in stress granuli formation and in the apoptotic pathway, promoting cell proliferation and migration in CRC cells, boosting also tumor growth in xenograft mice.
3. *Resistance to cell death*. It has been reported that miR-29 is an endogenous regulator of MCL-1 protein expression, an anti-apoptotic molecule, and it has been found down-regulated in cholangiocarcinoma cell lines (Mott et al., 2007). Similarly, miR-15a and miR-16-1, found deleted or down-regulated in the majority of chronic lymphocytic leukemias (CLLs), can directly negatively regulate BCL-2 in CLL. Their expression was described as inversely correlated to BCL2 expression in CLL and their over-expression may induce apoptosis in a leukemic cell line model through BCL2 repression (Cimmino et al., 2005).
4. *Enabling replicative immortality*. miR-130b~301b cluster is hypermethylated in prostate cancer cells and it was demonstrated how its expression restoration can replace senescence mechanisms reducing the malignant phenotype of prostate cancer cells (Chen et al., 2015; Ramalho-Carvalho et al., 2017). Similarly, miR-137 levels are significantly reduced in human pancreatic cancer leading to a defective senescence response. This small non-coding RNA targets KDM4A which expression contributes to avoid miR-137-induced senescence. Therefore, restoration of miR-137 expression it has been reported to promote senescence and dampen proliferation of pancreatic cancer cells (Neault et al., 2016).
5. *Inducing angiogenesis*. miR-135a is generally decreased in gastric cancer tissues compared to normal samples. It targets FAK which is an important regulator and effector of VEGF in tumor angiogenesis. It has been described that upon miR-135a over-expression, the protein levels of FAK in gastric cancer cell lines decrease significantly. Therefore, it has been proposed that miR-135 inhibits tumor growth, migration, invasion and angiogenesis by targeting focal adhesion kinase (FAK) pathway (Cheng et al., 2017). Differently, miR-23 in lung cancer cells under hypoxic conditions is up-regulated in the secretome and directly targets prolyl hydroxylase 1 and 2, enhancing the accumulation of the hypoxia-inducible factor-1α. Consequently, hypoxic lung cancer cells enhanced angiogenesis. In addition, it has been shown how secreted miR-23a also inhibits tight junction protein ZO-1, thereby increasing vascular permeability and cancer trans-endothelial migration. Moreover, inhibition of miR-23a dampened angiogenesis and tumor growth in mice and miR-23a found in sera of lung cancer patients positively correlated with proangiogenic activities (Hsu et al., 2017).
6. *Activation of invasion and metastasis*. Daugaard and colleagues demonstrated via RNA-seq analysis of formalin-fixed paraffin embedded (FFPE) lung adenocarcinomas from patients with and without detectable metastasis disease, that down-regulation of miR-30a-3p and up-regulation of miR-210-3p were significantly associated with the presence of distant metastases (Kumarswamy et al., 2012; Daugaard et al., 2017). Microarray analysis and quantitative PCR by the Law laboratory identified and validated up-regulated miR-885-5p in liver metastases when compared to primary CRCs. Furthermore, over-expression of miR-885-5p *in vitro* led to cell migration, invasion and *in vivo* development of liver and lung metastases. miR-885-5p targets directly the 3′UTR of CPEB2 which negatively regulates TWIST1, a well-known player in epithelial-mesenchymal transition (EMT) (Siu-Chi Lam et al., 2017). Alike, miR-9 may promote ovarian cancer metastasis targeting E-CADHERIN and upregulating N-CADHERIN and VIMENTIN, mesenchymal markers (Zhou et al., 2017).
7. *Reprogramming energy metabolism*. It is well-known that cancer cells are able to modify its metabolism favoring survival and proliferation. miR-7 has been demonstrated to decrease the usually up-regulated metabolic autophagy in pancreatic cancer cells via affecting LKB1-AMPK-mTOR signaling (Gu et al., 2017). Another tumor suppressor microRNA is miR-1 which has been described to be down-regulated in CRC cell lines compared to normal colon epithelial cells. Moreover, over-expression of miR-1 decreases cancer cell proliferation dampening aerobic glycolysis, lactate production and glucose uptake *in vitro* targeting HIF-1α and impacting SMAD3 pathway (Xu et al., 2017). On the contrary, high levels of miR-150 in glioma cells increased the Warburg effect, via the targeting of VHL 3′UTR, facilitating *in vivo* tumor growth (Li et al., 2017).
8. *Evading immune destruction*. Khorrami and colleagues showed how over-expression of miR-146 in a CRC cell line co-cultured with peripheral blood mononuclear cells extracted from healthy donors, increased T_reg_ frequencies and anti-inflammatory cytokines like TGF-β and IL-10, leading to an overall immune suppression in the tumor microenvironment (Rusca and Monticelli, 2011; Khorrami et al., 2017). On the other hand, miR-152 was shown to be decreased in gastric cancer cell lines as well as in human gastric cancer tissues. Restoration of its expression leads to enhanced T cells proliferation and effector cytokines production through the inhibition of the B7-H1/PD-1 pathway (Wang Y. et al., 2017).

**Table 1:** Examples of microRNAs involved in the hallmark of cancer.

**Hallmark**	**microRNA**	**De-regulation in cancer**	**Target**	**Function**	**References**
Sustaining proliferative signal	miR-27-3p	↑ in nasopharyngeal cancer	MAPK10	Regulation of ERK1 and ERK2 cascade	Li and Luo, 2017
	miR-545	↓ in colorectal cancer	EGFR	Signaling pathway	Huang and Lu, 2017
Evading tumor suppressors	miR-19a	↑ in colorectal cancer	TIA1	Major granule associated species	Liu Y. et al., 2017
Resistance to cell death	miR-29	↓ in cholangiocarcinoma	MCL-1	Regulation of apoptosis vs. cell survival, and maintenance of viability	Mott et al., 2007
	miR-15a, miR-16-1	↓ in chronic lymphocytic leukemias	BCL-2	Suppresses apoptosis	Cimmino et al., 2005
Enabling replicative immortality	miR-130b~301b cluster	↓ in prostate cancer	MMP2	Matrix remodeling	Ramalho-Carvalho et al., 2017;Chen et al., 2015
	miR-137	↓ in pancreatic cancer	KDM4A	Histone demethylase	Neault et al., 2016
Inducing angiogenesis	miR-135a	↓ in gastric cancer	FAK	Non-receptor protein-tyrosine kinase	Cheng et al., 2017
	miR-23	↑ in lung cancer	PH1; PH2; ZO-1	Alanine-Glyoxylate Aminotransferase; Glyoxylate And Hydroxypyruvate Reductase; Tight Junction Protein	Hsu et al., 2017
Activation of invasion and metastasis	miR-30a-3p	↓ in lung cancer	SNAI1	Induction of the epithelial to mesenchymal transition, growth arrest, survival and cell migration	Kumarswamy et al., 2012
	miR-885-5p	↑ in liver cancer	CPEB2	Cell cycle progression	Siu-Chi Lam et al., 2017
	miR-9	↑ in ovarian cancer	E-CADHERIN	Calcium-dependent cell adhesion	Zhou et al., 2017
Reprogramming energy metabolism	miR-7	↓ in pancreatic cancer	LKB1	Cell metabolism, cell polarity, apoptosis and DNA damage response	Gu et al., 2017
	miR-1	↓in colorectal cancer	HIF1α	Activation of genes involved in metabolism, angiogenesis, erythropoiesis and glycolysis	Xu et al., 2017
	miR-150	↑ in glioma cells	VHL	Regulates the hypoxia inducible protein HIF in normoxic conditions	Li et al., 2017
Evading immune destruction	miR-146	↑ in colorectal cancer	IRAK1; TRAF6	Initiates innate immune response against foreign pathogens; activation of NFKB by TNFRSFs	Rusca and Monticelli, 2011; Khorrami et al., 2017
	miR-152	↓ in gastric cancer	B7-H1	Costimulatory signal, essential for T-cell proliferation and production of IL10 and IFNG	Wang Y. et al., 2017

In this network of complexity, it should be added that one microRNA, in view of its target promiscuity, could have multiple roles in different type of cancers. miR-21 is one of those microRNAs. It has been found to be an anti-apoptotic factor in breast cancer (Si et al., 2007) and its suppression increased CASPASE3/7 enzymatic activities in human glioblastoma cells (Chan et al., 2005). Moreover, miR-21 is able to sustain proliferative signal targeting PTEN, a well-known tumor suppressor, in cholangiocarcinoma (He Q. et al., 2013; Wang L.-J. et al., 2015) and human hepatocellular carcinoma (Meng et al., 2007), inhibiting AKT and mTOR pathway which promotes cell survival and proliferation. miR-21 is linked with PI3K/AKT pathway also via the inhibition of FOXO1 in large B-cell lymphoma (Go et al., 2015). It was also shown to target TPM1 which normally is considered a tumor suppressor gene, regulating microfilament formation and anchorage-independent growth in a breast cancer cell line (Zhu et al., 2007). In addition, in breast cancer, miR-21 has been reported to sustain EMT signaling and IL-6 levels affecting the tumor immune microenvironment (De Mattos-Arruda et al., 2015). It is also true that some microRNAs may have a dual role in different cancer types, acting as tumor suppressor or onco-miR. miR-181a when overexpressed, was described in human glioma cells to induce apoptosis and dampen cell invasion (Shi et al., 2008) and migration in non-small cell lung cancer (NSCLC) (Cao et al., 2017). Interestingly, in human gastric cancer cells, miR-181a has been reported to be an onco-miR, promoting cell proliferation, wound healing invasion and EMT targeting RASSF6 (Mi et al., 2017). Thus, the complexity of the involvement of microRNAs in cancer is high and disentangling the dense net of RNAs interaction in order to build a complete and clear scenario will be a real challenge.

## MicroRNA as cancer biomarkers

As we have mentioned, it has become evident that microRNAs are involved in many aspects of cancer and because of their mechanism of action they control a big network of targets rather than few specific genes. This means that profiling microRNAs may give insights on complex processes hidden in numerous target genes, helping researchers to find new useful biomarkers. The definition of biomarker evolved with time and is not unique, but it could be summarized as “a characteristic that is objectively measured and evaluated as an indicator of normal biological processes, pathogenic processes, or pharmacologic responses to a therapeutic intervention” (Strimbu and Tavel, 2010). As a matter of fact, microRNAs possess most of the characteristics of the ideal biomarker, considering analytical criteria and clinical utility. They are specific to the pathology of interest, a reliable indication of the disease before clinical symptoms appear and sensitive to physiological or pathological changes. First demonstrations of the ability of microRNA expression patterns to be classifiers came in the first decade of 2000. Lu and colleagues implemented a bead-based microRNA profiling method in order to assess microRNA expression in normal and tumor tissues. Unexpectedly, they observed that precise pattern of microRNAs expression can, not only distinguish tumor origin, but also the degree of differentiation and classify poorly undifferentiated tumor tissues (Lu et al., 2005). Other evidences came from microRNA signatures that could discriminate between lung tumor tissues and correspondent non-tumor tissues. Differential expression was also seen between adenocarcinoma (AD) and squamous cell carcinoma (SCC) tissues and between distinct prognosis (Yanaihara et al., 2006). A wider analysis on 22 different types of tumor tissue, revealed a signature of 48 microRNAs able to reach a classification accuracy >90% (Rosenfeld et al., 2008). It is important to mention how NGS (Next Generation Sequencing) technologies revolutionized this field becoming progressively fundamental tools for personalized medicine (Schweiger et al., 2011). Even in microRNA studies these methods revealed completely new information which probably would not have been unveiled with standard techniques. In view of the big amount of data coming from NGS, new biomarkers have been discovered starting with an agnostic discovery platform methodology (Leidner et al., 2012; Wu et al., 2012) allowing researchers to be unbiased on their findings. There are some limitations that come together with the power of NGS, like costs, time-consuming experiments and management of big amount of data.

Pattern of microRNAs expression may be used to classify sub-population of patients in order to choose the right strategy in the clinical practice. However, we have to be aware that the microRNA signatures as biomarkers are not always due to a direct biological mechanism, but also to indirect specific consequence of the disease. In the following paragraphs, we report some examples (Table 2).

**Table 2:** **(part 1)** Examples of microRNAs as biomarkers.

**Type of classification**	**microRNA**	**Type of cancer**	**Reference**
Cancer VS Healthy	↑ miR-21	Breast cancer; lung cancer; stomach cancer; prostate cancer; colon cancer; pancreatic cancer; ovarian cancer; esophagus cancer; Ewing's sarcoma; liposarcoma; Wilm's tumor; osteosarcoma; oral tongue cancer	Iorio et al., 2005, 2007; Volinia et al., 2006; Markou et al., 2008; Hezova et al., 2015; Kapodistrias et al., 2016; Parafioriti et al., 2016; Calatayud et al., 2017; Chen et al., 2017; Cui et al., 2017; Nakka et al., 2017
	↑ miR-21, miR-96, miR-183, miR-182, miR-141, miR-200a, miR-429;	Breast cancer	Xiong et al., 2017
	↓ miR-139 and miR-145		
	↓ miR-424, miR-326, miR-511, miR-125b-2, miR-451	Hepatocellular cancer	Lu et al., 2017
Sub-typing	Panel of 63 microRNAs	Basal and luminal muscle-invasive bladder cancer	Ochoa et al., 2016
	Panel of 137 microRNAs	Basal and luminal A breast cancer	Blenkiron et al., 2007
	Panel of 19 microRNAs	Pancreatic ductal adenocarcinoma	Namkung et al., 2016
	miR-15a, miR-22, miR-141, miR-497, miR-129-5p, miR-185, miR-409-3p, miR-409-5p and miR-431-5p, miR-129	Lung neuroendocrine cancer histotypes	Rapa et al., 2015
	miR-21, miR-205, miR-375	Lung adenocarcinoma and squamous cell carcinoma	Lebanony et al., 2009; Del Vescovo et al., 2011; Patnaik et al., 2015
Cancer progression	↓ let7	Lung cancer	Takamizawa et al., 2004
	miR-221 and let7a protective, while miR-372 and miR-182-3p risky	Lung cancer	Yu et al., 2008
	Panel of 20 microRNAs	Lung cancer	Yanaihara et al., 2006
	↓ miR-448	Lung cancer	Shan et al., 2017
	↓ miR-383	Lung cancer	Shang et al., 2016
	↑ miR-187	Lung cancer	Peng et al., 2016
	↓ miR-187	Renal cell carcinoma	Zhao et al., 2013
	↓ miR-187	Ovarian cancer	Chao et al., 2012
Cancer Therapy	↑ miR-21	Colon cancer (poor fluorouracil based adjuvant chemotherapy outcome)	Schetter et al., 2008, 2009
	↑ miR-21	Pancreatic cancer (poor fluorouracil-based adjuvant chemotherapy outcome)	Hwang et al., 2010
	↑ miR-21	Lung cancer (poor platinum-based chemotherapy outcome)	Gao et al., 2012
	↑ miR-448	Lung cancer (cisplatin resistance)	Fang et al., 2016, 2017
	↓ miR-138	Lung cancer (cisplatin resistance)	Wang et al., 2011
	↓ miR-10b	Pancreatic cancer (highly predictive response to gemtabicine-based multimodality neoadjuvant therapy)	Preis et al., 2011
	↓ miR-148	Colorectal cancer (poor fluorouracil and oxaliplatin-based therapy outcome)	Takahashi et al., 2012
	miR-221, miR-222, miR-331, miR-451, miR-28, miR-151, miR-148a, miR-93, miR-491	Diffuse large B-cell lymphoma (prediction of OS and PFS after rituximab and chemotherapy treatment)	Montes-Moreno et al., 2011
	↑ miR-31-3p	Colorectal cancer (poor anti-EGFRmAb therapy outcome)	Mosakhani et al., 2012
	↓ miR-592		
	↑ signature of let7c, miR-99a, miR-125b	Colorectal cancer (good cetuximab and panitumumab outcome)	Cappuzzo et al., 2014
	↑ miR-31-3p, miR-31-5p	Colorectal cancer (lower PFS after anti-EGFRmAb therapy)	Igarashi et al., 2015; Mlcochova et al., 2015
	↑ miR-200c	Lung cancer (good of EGFR-TKIs therapy outcome)	Li et al., 2014b
	A panel of 29 microRNAs	Renal cell carcinoma (TKIs therapy outcome)	Garcìa-Donas et al., 2016
	miR-181a-5p, miR-339-5p	Hepatocellular carcinoma (prediction of sorafenib therapy outcome)	Nishida et al., 2017
	↑ miR-183	Renal cancer (less efficacious cancer cytotoxicity by natural killer cells)	Zhang et al., 2015
	↑ miR-6826, miR-6875	Colorectal cancer (poor vaccine therapy outcome)	Kijima et al., 2016

*From diagnosis to the choice of therapeutic intervention*.

### Biomarkers for cancer diagnosis and sub-typing

Biomarkers can stratify patients upon different aims. One of the first clinical questions could be to understand whether the physician is facing a pathological condition. Therefore, discriminating between tumor tissues and non-tumor tissues is extremely important. The most recurrent example is miR-21 which is over-expressed in many cancer types (Iorio et al., 2005, 2007; Volinia et al., 2006; Markou et al., 2008; Hezova et al., 2015; Kapodistrias et al., 2016; Parafioriti et al., 2016; Calatayud et al., 2017; Chen et al., 2017; Cui et al., 2017; Nakka et al., 2017). The problem of using such microRNA as a biomarker is the absence of specificity. Therefore, signatures of a pattern of microRNAs are generally preferred to deliver a specific diagnosis. A nine microRNAs signature was able to discriminate between breast cancer tissues and normal cancer tissues collected by TCGA, with a high accuracy value and AUC of 0.995 (Xiong et al., 2017). Another example comes from the He group which found five microRNAs (miR-424, miR-326, miR-511, miR-125b-2 and miR-451) able to provide high diagnostic accuracy of hepatocellular carcinoma starting from microRNA expression profiles of 377 hepatocellular carcinoma patients (Lu et al., 2017). As finding the pathological condition is relevant, the step forward is to understand what type of condition the clinician is facing. It is well-known that each cancer type is composed of several subtypes coming from different cellular origins and each of them has to be treated accordingly. Thus, it is important to discriminate among them and several studies pointed at this aim. A study on muscle-invasive bladder cancer in 2016 revealed a signature of 63 microRNAs able to discriminate between basal and luminal tumors and a 15 microRNAs based signature able to show basal and luminal tumors with apparent fibroblast infiltration (Ochoa et al., 2016). Similarly, Blenkiron and colleagues performed a model-based discriminant analysis for basal-like and luminal A breast tumors finding a set of microRNAs able to discriminate between those groups (Blenkiron et al., 2007). Another approach by the Jang lab exploited the expression of 1,733 microRNAs to build an unsupervised clustering in order to distinguish subtypes of pancreatic tumors. As result, they found 3 subtypes which could be associated with patient prognosis (Namkung et al., 2016). In lung cancer, in the data of the Volante lab, 10 microRNAs were able to distinguish between lung neuroendocrine (NE) tumors histotypes, 9 of which also discriminated between carcinoids and high-grade NE carcinomas (Rapa et al., 2015). In addition, combination of miR-21 and miR-205 was found to be able to distinguish lung AD from SCC (Lebanony et al., 2009) and this can be further improved with the analysis of miR-375 (Patnaik et al., 2015). As a matter of fact, we demonstrated the non-perfect reliability of miR-205 in discriminating AD vs. SCC lung cancer histotypes (Del Vescovo et al., 2011).

### Biomarkers for cancer progression

Understanding the aggressiveness and progression of cancer via prognosis of the patient is of enormous relevance in clinical practice. microRNAs are able to predict patient prognosis in several types of cancer. Here show some examples from lung cancer. Let7 was found to be down-regulated in lung cancer *in vitro* and *in vivo*. A cohort of 143 lung cancer tissues was analyzed for the expression of let7 which resulted significantly down-regulated compared to normal tissues. Moreover, reduced let7 associates with higher disease stages and poor post-surgery survival and prognosis. Taking into account only the AD samples, these distinctions are maintained (Takamizawa et al., 2004). A wider analysis led to discover a 5 microRNAs signature (miR-221 and let7a protective, while miR-137, miR-372 and miR-182-3p risky) able to discriminate between NSCLC patients with higher or lower median overall survival (OS) independently from stage or histology. However, this signature is able to predict patient survival within histological type AD or SCC (Yu et al., 2008). With a similar strategy, a pattern of unique 15 microRNAs was able to discriminate between lung SCC and normal tissues, while a signature of 20 microRNAs was able to predict the OS (Raponi et al., 2009). Some of these microRNAs were more significant, like miR-146b which had the highest prediction score within 3 years, and some had already been linked to lung cancer in other studies like let-7 and miR-155 (Yanaihara et al., 2006). Interestingly, in all these studies, the different isoforms of let-7 found, were down-regulated in patients with poor prognosis. More recent data show that low expression of miR-448 associates with lung SCC progression and poor patients overall survival (Shan et al., 2017). Reduced expression of miR-383 was found in NSCLC tumor tissues compared to adjacent non-tumorous samples and moreover, low miR-383 expression associated with poor post-operative prognosis (Shang et al., 2016). miR-448 and miR-383 are down-regulated, acting like tumor-suppressors, also in ovarian cancer (Lv et al., 2015), hepatocellular carcinoma (Zhu et al., 2015; Chen et al., 2016), colorectal cancer (Li et al., 2016), breast cancer (Li et al., 2011), Hodgkin lymphoma (Paydas et al., 2016), glioma (He Z. et al., 2013; Xu et al., 2014) testicular carcinoma (Lian et al., 2010; Huang et al., 2014) and medulloblastoma (Li et al., 2013). Another study revealed that miR-187 expression was significantly increased in NSCLC tissue samples compared to adjacent non-lung tumor tissues and that this condition associated with TNM classification and shorter OS (Peng et al., 2016). Interestingly, miR-187 has been found down-regulated in clear cell renal cell carcinoma cells (Zhao et al., 2013) but up-regulated in ovarian cancer cells (Chao et al., 2012). However, in both cases the studies agree with what occurs in lung cancer, where low miR-187 level of expression is associated with poor patient survival.

### Biomarkers for cancer therapy

As a consequence of the intricacy of underlying driving mechanisms of cancer, therapeutic efficacy of a single treatment can change depending on the patient and its type of cancer. microRNAs have been associated to, and also predictive of, therapeutic outcome. Here we report cases of some of the main standard cancer treatments.

miR-21 seems to be a general signal for chemotherapy resistance. In 2008, Schetter and colleagues found that miR-21 expression, in typical colon AD from patients treated with fluorouracil based adjuvant chemotherapy, is higher in patients with a poor therapy outcome (Schetter et al., 2008, 2009). Similar results were obtained for pancreatic cancer (Hwang et al., 2010). Even in lung cancer, high-expression of miR-21 was associated with chemotherapy resistance in tissues of patients who had undergone platinum-based chemotherapy treatment (Gao et al., 2012). It was shown that A549/DDP lung AD cell line has a lower expression of eIF3a compared to its parental cell line, and it displays chemoresistance to cisplatin. miR-488 targets the 3′UTR of eIF3a transcript enhancing sensitivity to the treatment and inhibiting cell proliferation, migration and invasion (Fang et al., 2016, 2017). Another study reported an increased sensitivity of A549/DDP cells to cisplatin after up-regulation of miR-138. The excision repair cross-complementation group 1 (ERCC1) was targeted by miR-138 and the result was the down-regulation of the protein correlating with increased levels of miR-138 in A549/DDP cells (Wang et al., 2011). In another study on pancreatic ductal AD, patients with resectable or locally advanced disease showed relative low miR-10b expression associated with highly predictive response to gemtabicine based multimodality neoadjuvant chemoradiotherapy. Moreover, by logistic regression, low miR-10b expression was able to predict surgery efficacy. miR-10b levels demonstrated significant ability in survival prediction (Preis et al., 2011). In CRC, miR-148 expression had a potential for predicting therapeutic efficacy of 5-fluorouracil and oxaliplatin in patients with stage IV colorectal cancer, as low levels of this microRNA associated with bad therapeutic response (Takahashi et al., 2012). Sensitivity to cisplatin treatment is, at least partially, regulated by miR-488 which targets eIF3a.

Besides chemotherapy, targeted therapy is an important standard of care for several tumors. Even in this field, microRNAs may be helpful. For diffuse large B-cell lymphoma (DLBCL), combination between chemotherapy and immunotherapy with rituximab has become a standard treatment. A 9 microRNAs signature was able to predict both OS and progression free survival (PFS) in DLBCL patients (Montes-Moreno et al., 2011). In a cohort of metastatic colorectal cancer patients wild type for KRAS and BRAF, a miR-31-3p up-regulation and miR-592 down-regulation were found associated with poor response to anti-EGFRmAb (Mosakhani et al., 2012). An Italian study reported a signature of three microRNAs (miR-let7c, miR-99a, and miR125b) able to predict EGFR monoclonal antibody therapy outcome in colorectal cancer patients. Indeed, high-level of signature expression showed a good discrimination capacity for patients which were more responsive to cetuximab or panitumumab compared to low responsive patients (Cappuzzo et al., 2014). In two independent studies, miR-31 was found to be associated with PFS after administration of anti-EGFRmAb in metastatic colorectal cancer patients. Mlchocova and colleagues found both miR-31-5p and−3p, while Shinomura group only miR-31-5p, to be higher in patients with lower PFS compared to those with low levels of the microRNA (Igarashi et al., 2015; Mlcochova et al., 2015). microRNAs have been discovered to be predictive also of kinase inhibitors efficacy in hepatocellular carcinoma, renal cell carcinoma and NSCLC (Li et al., 2014b; Garcìa-Donas et al., 2016; Nishida et al., 2017).

An emerging field in cancer treatment is immunotherapy. Some studies describe microRNAs as biomarkers of immunotherapy efficacy. In a report on 82 renal cancer patients and 19 healthy individuals, miR-183 has been found up-regulated in sera associated to less efficacious cancer cytotoxicity by natural killer cells, which are the effectors of the IL-2 immunotherapy (Zhang et al., 2015). Nagano group described that miR-6826 and miR-6875 can be good predictor of vaccine treatment efficacy in metastatic CRC, where high expression in plasma of two microRNAs was associated with poorer prognosis (Kijima et al., 2016).

## Circulating microRNAs

Over the last two decades, it has been demonstrated that a substantial number of microRNAs are present outside cells in blood and other body fluids, the so-called “circulating microRNAs” (c-microRNAs). C-microRNAs have been reported to be very stable under harsh conditions and able to survive high temperatures, extreme pH, and RNase activity. As reviewed by Makarova and colleagues (Makarova et al., 2016) they are often found in association with small membranous particles (extracellular vesicles) and mostly with RNA-binding proteins (Ago2, HDL, etc.). The extracellular vesicles (EV) are represented by a various population of membranous particles with different origins and sizes. Microvesicles originate through the budding of the plasma membrane and have a size around 100–1,000 nm. Exosomes, around 40–100 nm in size, are generated after the fusion of multivesicular bodies with the plasma membrane. The presence of these different carrier options leads researchers to think of selective microRNA sorting and secretion processes, not excluding stochastic (non-selective) ones. Moreover, the pool composition of the microRNAs is different intra- and extracellularly. Unfortunately, the exact mechanisms underlying these processes have to be discovered yet. Even though extracellular vesicles biogenesis is varied, only ceramide-dependent mechanism has been reported as one of the responsible for microRNA secretion so far (Kosaka et al., 2010). About sorting, it has been suggested that the affinity between the RNA and the raft-like membrane regions of the multivesicular bodies (MVBs) can guide it (Janas et al., 2015). In another study, Squadrito et al. (2014) reported that sorting of microRNAs to exosomes is partially regulated by the changes in expression of the targets inside the cell. The finding of a different microRNA sorting in exosomes depending on the KRAS status (Cha et al., 2015), adds concrete value on the selective sorting hypothesis. In 2013, Sànchez-Madrid group (Villarroya-Beltri et al., 2013) performed several microarrays analyses of activation-induced changes in the microRNA and mRNA profiles among T-lymphoblasts and their exosomes. They obtained a discordance between intracellular and extracellular microRNA and mRNA pool composition, demonstrating once again that the sorting into exosomes is not—at least completely—passive. Interestingly, they reported a short sequence motif (GGAG) enriched in exosomal microRNAs. Among the many heterogeneous nuclear ribonucleoproteins (hnRNPs) that precipitated with intracellular and exosomal microRNAs, only hnRNPA1 and hnRNP2B1 seemed to bind exclusively the latter. Another study demonstrated that Vps4A, a key regulator of exosomes biogenesis, seemed to regulate the sorting of oncogenic and oncosuppressive microRNAs in exosomes, favoring the inclusion of the first ones (Wei et al., 2015). Furthermore, it has been described how, in B cells, 3′end adenylated microRNAs appear to be enriched in cells compared to 3′ end uridylated isoforms which are more present in exosomes (Koppers-Lalic et al., 2014). What remains really unclear is the mechanism of sorting, if present, and of secretion of AGO-microRNA complexes. To date, the study reported by Turchinovich and colleagues, leads to think that the majority of these complexes is freed in a non-selective manner, because of the positive correlation between the content of c-microRNA in culture media and the increase of cell death (Turchinovich et al., 2011). Moreover, the Cayota group described via RNA-seq analysis, how expression values of individual microRNAs in intracellular fractions of MCF-7 cells after a certain threshold, correlated directly with extracellular values, suggesting a passing mechanism of release also for extracellular vesicles related microRNAs (Tosar et al., 2015). However, these conclusions do not wipe out at all the possibilities of a parallel selective secretion.

The presence of putative precise processes underlying c-microRNAs suggests that they could have an intriguing role in cell-cell communication. For instance, it was demonstrated that microRNAs enriched in extracellular vesicles derived from bone marrow mesenchymal stem cells can be absorbed by tubular epithelial cells resulting in the inhibition of expression of the known targets (Collino et al., 2010). Furthermore, as it has been reviewed (Neviani and Fabbri, 2015), c-microRNAs can influence cancer cells and their surrounding environment both targeting mRNAs and functioning as receptor-like systems. Much of the data supporting this way of cell-cell communication is done through *in vitro* systems, pushing for new validating studies *in vivo* which may confirm this hypothesis. One of the unclear point which can be argued is whether the actual amount of c-microRNAs is enough to drive expression changes in recipient cells *in vivo*. Some studies report that the average amount of microRNAs in exosomes is about 1 unit per exosome (Chevillet et al., 2014; Guzman et al., 2015). This very low amount may lead to some skepticism around the role of c-microRNAs in cell-cell communication. However, extracellular vesicles associated microRNAs are a small percentage of the total pool of c-microRNAs (Arroyo et al., 2011) and in addition, this semi-quantitative reasoning is far too simplistic, not taking into account, for example, the accumulation of microRNAs in recipient cells and, being a median measure, doesn't consider the content heterogeneity of extracellular vesicles.

Not only microRNAs from tissue can be used to create pattern of signatures able to classify group of patients but also c-microRNAs from liquid biopsies are becoming an increasing source of information (Chen et al., 2008). c-microRNAs as biomarkers have some advantages like great stability, resistance to ribonucleases and to severe physicochemical conditions in body fluids, increasing the feasibility of their use in clinical applications (Mitchell et al., 2008). Another important aspect is the compliance of the patients. Indeed, c-microRNAs are extracted from several body fluids coming from liquid biopsies, which are much less invasive and painful for the patients compared to the standard methods. Moreover, the cost and time for the processing is lower than non-liquid samples, promising big step toward the implementation of personalized medicine. Thus, the interest of the scientific community has grown intensively as demonstrated by the number of articles published in recent years at the entry “circulating microRNAs cancer biomarker” on PubMed (2012 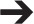 86; 2013 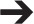 148; 2014 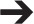 188; 2015 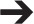 239; 2016 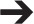 201). Moreover, there are some reviews which try to collect as much as possible the huge amount of information on circulating microRNAs as cancer biomarkers (Del Vescovo and Denti, 2015; Armand-Labit and Pradines, 2017; Matsuzaki and Ochiya, 2017; Zhao et al., 2017).

## MiRSNP

**Figure 2:**
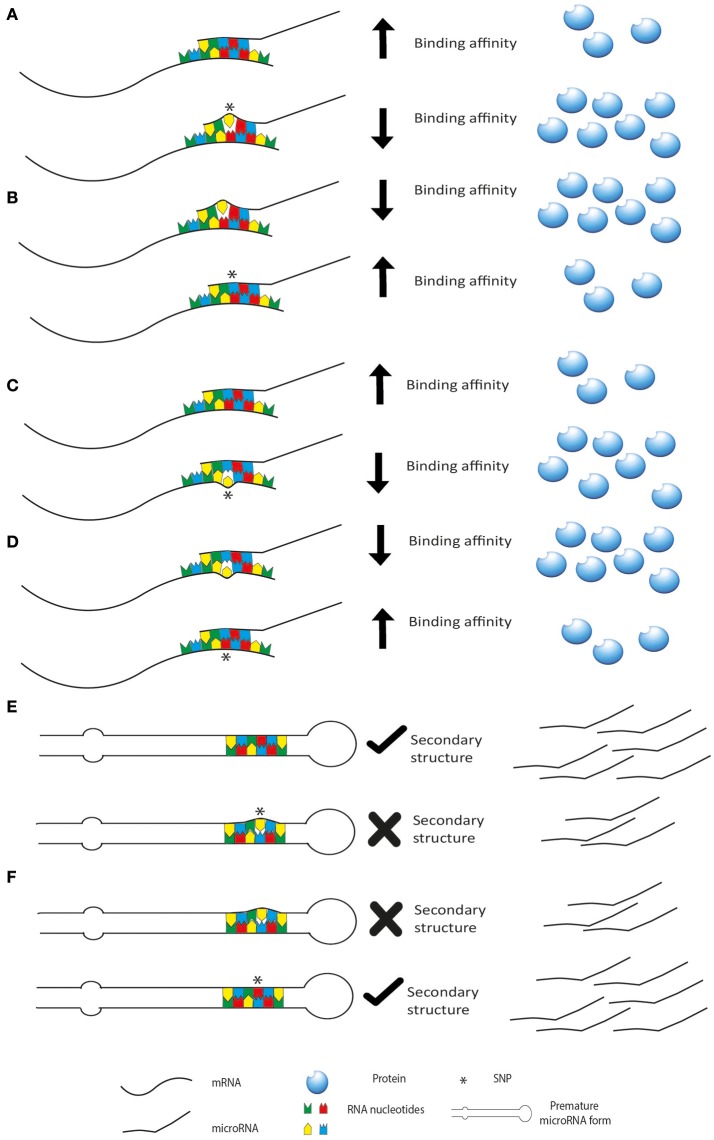
miRSNP can affect microRNA biogenesis and activity. SNPs may be present on the microRNA decreasing **(A)** or increasing **(B)** its binding affinity for the target mRNA. SNPs may be present in the binding site of a target mRNA decreasing **(C)** or increasing **(D)** binding affinity (or creating new binding sites). In this last scenario are represented also SNPs in genes of the microRNA biogenesis machinery. These SNPs usually affect the regulation of the genes increasing or decreasing binding affinity of post-transcriptional regulators like microRNAs. SNPs may also affect the secondary structure of premature forms of the microRNAs decreasing **(E)** or increasing **(F)** their maturation.

microRNAs exert their function through an interaction with seed sequence on either 3′UTR, 5′UTR or the coding sequence of a target mRNA. The hybridization between the two RNAs follows the Watson-Crick base pairing rules and thus it is guided by the formation of a stabilized double strand structure. Thus, when even single nucleotide changes occur in the sequence of either of the two interactors (miRSNP), the stability of the contact is affected and so is the functional outcome. Indeed, single nucleotide polymorphisms (SNPs) can affect microRNA expression and function and they can be present in the sequence of the microRNA or on its target genes (Figure 2). SNPs may be present also in the sequence of genes involved in the biogenesis of microRNAs, thus affecting their level of expression. These changes can influence the above-mentioned patterns of microRNAs, creating completely new classes of patients based on association to risk of cancer or prediction to therapy. Moreover, as reviewed by Del Favero group, it should be considered that SNP density is higher in the flanking region of the microRNA sequence compared to microRNA genes themselves and mature form of microRNAs has lower SNP density than the pre-microRNA. Interestingly, the seed sequence has the lowest SNP density, highlighting their evolutionary and functional importance (Cammaerts et al., 2015).

In literature, there are numerous reports describing cancer risk association with SNPs related to microRNAs life. In a meta-analysis by Liu and colleagues (Liu H. et al., 2017), conducted on ten studies with 6,000 cases and 7,664 controls, a significant association of miR-608 rs4919510 polymorphism with decreased cancer risk via recessive model (CC vs. GG + GC) was found. Interestingly, the same polymorphism has been already described to predict the clinical outcome in patients with different cancer types (Lin et al., 2012; Zheng et al., 2013; Pardini et al., 2015). As mentioned above, SNPs associated with microRNAs can alter several of their usual processes. One of the most common alteration is the degree of target suppression. rs73239138 polymorphism in miR-1269 was associated with increased susceptibility to hepatocellular carcinoma (HCC) and HBV-related HCC in a positive dominant model where genotypes with the A allele increased the risk to cancer (Min et al., 2017). It was shown that in HCC cell lines the over-expression of the miR-1269 variant led to a decreased inhibition of cell growth compared to the over-expression of the wild type microRNA. The authors confirmed the biological outcome demonstrating that the polymorphism on miR-1269 produced a dampened suppression of pErK1/2, SPATS2L and LRP6 compared to the wild type variant, where the last two genes showed to have a 3′UTR binding site for miR-1269. In a study on an Indian population (Sibin et al., 2017), miR-196a2 expression varied with age, tumor grade and tumor type among glioma patients' tissues but not with different genotype of the microRNA. However, they found a significant difference in the expression of its mRNA target HOXC8 depending on different genotypes where CC and TT showed decreased and increased expression, respectively. Remarkably, out of 72 sample pairs of tumor tissues and blood samples, 19,44% showed different genotype of miR-196a2 in the tissue compared to the blood suggesting a critical role in tumorigenesis and in changing of tumor grade. Other evidences pointed out the importance of polymorphisms altering the ability of the microRNAs to post-transcriptionally inhibit gene expression of their target in colorectal (Liu Y. et al., 2016) and gastric cancer (Liu C. et al., 2016). Along with a negative effect of a SNP on microRNA sequence, this variation may lead also to positive effects, increasing binding capacity of the microRNA to its target (Gong et al., 2012). To the best of our knowledge, there are no reports of this scenario in cancer yet, neither *in vitro* nor *in vivo*.

Undoubtedly, variations in the sequence of microRNAs can also affect their maturation. Bioinformatic analysis of Gibbs free energy on the structure of the miR-146a stem loop showed that the G allele of the rs2910164 polymorphism increased the stability factor of the overall structure, suggesting that, the association between the C carrier allele in the Iranian population under study may correlate with lower expression of miR-146a and thus higher incidence of the presence of its target Her2 in breast cancer (Meshkat et al., 2016). Indeed, miR-146 is significantly higher in triple-negative breast cancer compared to non-triple-negative breast cancer (Garcia et al., 2011). The already mentioned polymorphism on miR-196a2 was associated in other cancer studies to the microRNA maturation, changing the miR-196a2 expression (Hu et al., 2008, Hoffman et al., 2009). Another common scenario is the presence of a polymorphism in the microRNA binding site of an mRNA target. In a study on 325 colorectal cancer (CRC) patients and 977 normal individuals, the polymorphism rs7930 in the 3′UTR of TOMM20 was found to be associated with CRC susceptibility and the G allele described as the risk allele. Via *in silico* target analyses, miR-4273-5p was predicted to interact with rs7930. Validation by luciferase assay in human CRC cell lines demonstrated that the G allele plasmid did not have any effect on the reporter compared to a stronger effect of the A allele plasmid. Moreover, cell lines with the AA genotype showed a considerably stronger dampen in TOMM20 levels than those with the AG genotype (Lee et al., 2016). On two stages study made of 2347 cases and 3390 controls in total, Ke and colleagues found that the polymorphism rs1062044 on the sequence of LAMC1 produces an increased risk of colorectal cancer in the GA genotype compared to GG. Moreover, it decreases the ability miR-423-5p to bind LAMC1 in CRC cancer cell lines (Ke et al., 2017). The presence of a polymorphism on the binding site could also lead to a positive effect, thereby creating a new illegitimate binding site. Bartel group found that polymorphism SNP34091 in the 3'-UTR of MDM4 creates a new binding site for miR-191 in ovarian cancer (Wynendaele et al., 2010).

Polymorphisms on genes involved in the microRNA biogenesis can also have an impact on cancer progression. Mullany and colleagues, through RNA-seq and GWAS analysis of colon cancer tissues, found 24 microRNAs which were deregulated in the presence of SNPs significantly associated with altered mRNA expression or cancer risk. In particular, rs2740349 (GEMIN4) and rs235768 (BMP2) were shown to be associated with microRNA expression variation, with up-regulation corresponding to the variant genotypes (Mullany et al., 2016). Interestingly, this up-regulation is associated with a down-regulation of the mRNAs of biogenesis genes, implicating new roles for these genes or other mechanisms of microRNA expression influence. In addition, Rotunno and colleagues found that RNASEN/rs640831, present in the GTACCT haplotype was associated with variation in expression of 56 microRNAs, both up- and down-regulated (Rotunno et al., 2010). One mechanism of action of these SNPs is the change in binding affinity of a regulator of the transcript like a microRNA. Jiang and colleagues genotyped 24 SNPs in a cohort of 878 breast cancer patients and 900 controls. They found that polymorphism rs417309 is associated with higher breast cancer risk (Jiang et al., 2013). Moreover, this SNP is placed on the 3′UTR of the DGCR8 mRNA, affecting the binding ability of miR-106b and miR-579. However, the imperfect relationship between up-/down-regulation of microRNA biogenesis-related genes and up-/down-regulation of microRNAs, suggests a series of effects which are far to be completely clear and understood.

Behavioral changes of microRNAs upon SNPs can also affect the performance of cancer drugs. Pharmacogenomics studies how single genome or transcriptome variations can affect pharmacokinetics (PK) and dynamics (PD). A lot of attention has been drawn to microRNAs as possible players in this area. The contribution of microRNAs to PK and PD has been studied also via bioinformatic tools. Some genes are specific to PK, some to PD, whereas others to both. *In silico* data hint to a higher relevance of post-transcriptional regulation by microRNAs on PD unique genes compared to PK unique genes (Rukov et al., 2011). As a matter of fact, the latter show on average a shorter 3′UTR with a less dense presence of predicted target binding site for microRNAs, compared to the former. Among some genes coding for drug metabolizing enzymes (DMEs), transporters and nuclear receptors, CYP1A2, CYP2B6, CYP2D6, CYP3A4, NR1I2, and UGT2B7 were sequenced for their 3′UTR in a population of 30 South Africans (Swart and Dandara, 2014). 40 out of 52 SNPs detected were predicted to potentially create or abolish microRNA binding sites, thus affecting regulation capacity and expression of those genes. Despite the low number of patients enrolled, this study highlights once more the engagement of microRNAs in pharmacogenomics. Therefore, an increasing interest in creating web tools to analyze miRSNP and drugs has grown.

Mir2Drug is a database able to calculate the influence of miRSNP in drug efficiency. It considers the sequence 30 bp up- and downstream a known SNP in the 3′UTR of target genes, and calculates all the predicted binding sites for microRNAs in that region, analyzing the change in free energy from wild type to variant genotype. Upon significant differences, Mir2Drug associates these SNPs as either direct or indirect drug targets. Therefore, it provides comprehensive annotation information on miRSNP belonging to drug target genes (Wang X. et al., 2017).

SMiR-NBI is another bioinformatic tool available on the web, which provides insights on possible pharmacogenomic biomarkers characterized by microRNAs, comprehending a network connecting small molecules to microRNAs regulation (Li et al., 2014a). This growing interest on miRSNP and pharmacogenomics comes from several works and here we try to review examples from different cancer types. A standard of care therapy for advanced lung cancer patients is the platinum-based chemotherapy. This kind of therapy leads to a spectrum of toxicities with different degree of severity. A study of Fang et al. (2016) found that the polymorphism rs2042553 of miR-5197 significantly associates with severe toxicity after platinum-based treatment. Moreover, miR-605 polymorphism rs2043556 was associated with hepatotoxicity, while miR-27a rs895819 was related to gastrointestinal toxicity. Platinum-based therapies are often combined with other drugs like gemcitabine or paclitaxel. Geng et al. (2016) studied the effect of different regimen of chemotherapy based on cisplatin plus paclitaxel, gemcitabine or Changchun vinorelbine, in a cohort of advanced NSCLC patients. They found that polymorphism rs11077 in XPO5, a transport factor involved in the export of pre-microRNAs from the nucleus to the cytoplasm, is associated in AA genotype to a worse prognosis in a chemotherapy regimen compared to the AC genotype. Human Pregnane X Receptor (PXR) induces expression of DMEs, thus it can potentially influence the efficacy of several anticancer drugs. In a study on 96 Indian breast cancer patients (Revathidevi et al., 2016), genomic DNA from blood samples was sequenced for PXR 3′UTR. Among 12 SNPs already reported in several databases, 5 SNPs were observed and in particular, for SNPs rs3732360 and rs3732359 the proportion of the mutant allele is higher compared to the wild type in the studied population. These two polymorphisms conferred a new binding site for miR-500a-3p and decreased the binding of miR-532-3p which is known to play a role in doxorubicin cardiotoxicity (Wang J.-X. et al., 2015). In fact, the observed SNPs either created new binding sites for microRNAs, or abolished some of them, or strengthen or dampened the binding capacity of others. Therefore, overall regulation of PXR could be affected impacting on the metabolism of drugs. As a matter of fact, microRNA predicted to be influenced by these SNPs are also involved in treatment efficacy and doxorubicin cardiotoxicity, as pathway analysis revealed. Even in non-solid tumors microRNA variation may impact on treatment efficacy and toxicity. Lòpez-Lòpez and colleagues studied possible associations between miRSNP and adverse reactions after methotrexate administration in Acute Lymphoblastic Leukemia (ALL) (Lopez-Lopez et al., 2014). They showed that SNP rs639174 in DROSHA is associated with vomit during consolidation of methotrexate treatment, as well as rs56103835 in pre-miR-453. Moreover, rs12894467 in pre-miR-300 is associated with hepatic toxicity and hyperbilirubinemia in induction. However, the same group found that none of the miRSNP genotyped in a Spanish population of 152 ALL affected children, is associated with Vincristine-related neurotoxicity (Lopez-Lopez et al., 2016), highlighting how microRNAs are not involved randomly in every process, but they are selectively and directly responsible or indirectly involved in some divergent mechanism.

It is worth mentioning that also big sequence changes like INDELs in microRNAs related genomic regions can have an impact on how drugs are affected by human body and vice versa. Garcìa-Ortì et al. (2012) found 19 microRNAs associated with gene copy number variations in genomic regions where they are located, in acute myeloid leukemia (AML) cells. 4 out of 19 had NF1 as a potential target gene but only miR-370 was then validated. Patients analysis showed that NF1 down-regulation by either miR-370 over-expression or NF1 gene deletion is common in AML. Thus, considering that NF1 deficiency leads to RAS activation, patients with over-expression of miR-370 may potentially take advantage from RAS or mTOR inhibitors (Parkin et al., 2010). Another study (Bruhn et al., 2016), pinpointed that different lengths in the 3′UTR ATP binding cassette (ABC) membrane transporter P-gp (ABCB1) may alter the presence of several microRNA binding sites. Actually, imatinib resistant leukemia cell lines expressed shorter 3′UTR potentially losing some regulating sequences. Indeed, the shortening of ABCG2 (another ABC transporter) 3′UTR removes miR-519c binding site, therefore contributing to drug resistance (To et al., 2009).

## Limitations of microRNAs as tools for personalized medicine

microRNAs are intensively studied as tools for personalized medicine because they encompass many ideal characteristics for fast and robust analysis, which is needed in clinical practice. As a matter of fact, they are generally stable due to protein based carriers or EVs engulfment. Moreover, the detection is easier so far, considering the low amount required, the hybridization methods criteria which avoids the production of complex probes like antibodies for proteins and the accessibility of the technologies. The ability of microRNAs to fine tune the gene expression enables these markers to be more sensitive in the pathology follow-up. On the contrary, biomarkers like ctDNA (circulating tumor DNA), which is a promising new biomarker for cancer practice, being strictly linked to genomic mutation analysis, suffer from uncertainty in ongoing follow-up (Nadal et al., 2017). However, some problems still limit the use of microRNAs in personalized medicine. The source of microRNAs has to be managed accordingly and the influence on recovery and final outcome may be substantial, especially for RNA extracted from biofluids. Regarding the detection, the short sequence of microRNAs impedes an easy design of probes, limiting also the discrimination between pri-, pre- and mature forms. Moreover, despite the ease of use and accessibility of qRT-PCR, ddPCR, microarrays and NGS as main detection techniques for nucleic acids, they lack strong sensibility and accuracy, especially at single base resolution. Another key point is the normalization of the signal. In fact, as the analysis is about a relative expression, the choice of a good normalizer is fundamental and challenging (Masè et al., 2017). In addition to these analytical problems, the complex biology of microRNAs increases the obstacles toward a full comprehension of these small non-coding RNAs. As a matter of fact, functional studies with microRNAs suffer from absence of physiological conditions, thus when over-expression studies are performed it should be taken into account that microRNAs generally act with low quantities and more than one on a single target.

Indeed, personalized medicine is going through the use of c-microRNAs instead of tissue-derivatives. Despite the clear high potential of c-microRNAs in the future personalized medicine, technical difficulties to perform robust and comparable profiling of these small nucleic acids have impeded progress to develop an approved clinical diagnostic assay (Jarry et al., 2014).

**Figure 3:**
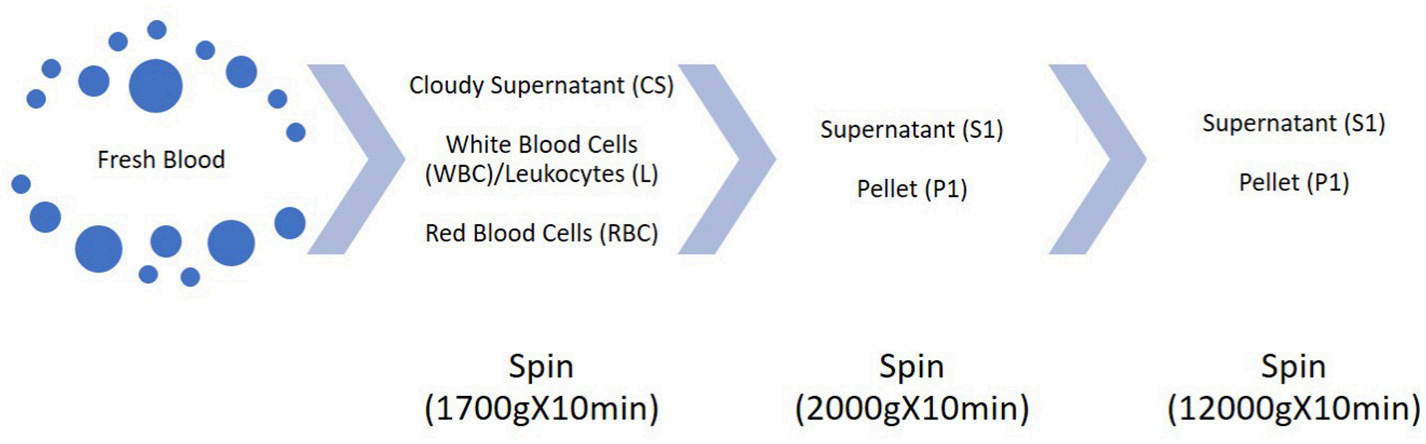
Scheme of the multiple centrifugation steps performed in Duttagupta et al. (2011).

The problems come through three different steps in the analysis of c-microRNAs: pre-analytical, analytical and post-analytical phase. Therefore, from where and how c-microRNAs are extracted, how we detect them and how we process the data, still leads to great variability among different studies. Pre-analytical variables are those factors which can affect the composition of the sample to be tested: from patient's conditions variability to sample handling. Firstly, it has to be considered that usually, c-microRNAs are in low titer in biofluids compared to microRNAs in sample tissues. Considering the study of Tewari and colleagues, the concentration of microRNAs in plasma can be counted as from 100 to 9,000 copies per uL or, as shown in another study with ddPCR, even up to 23,000 copies/uL (Miotto et al., 2014). Similar results were found for cardiac injury induced microRNAs (Thompson et al., 2016). Moreover, the lack of knowledge about the secretion and sorting of the microRNAs outside the cells, puts some limits on the patient's condition which would ensure reproducibility on the assays. Another challenge is represented by the contaminant microRNAs. It is known that c-microRNAs come from different cellular sources. Tewari group showed that blood cells are the major contributor to c-microRNAs, therefore variations in blood cells counts and hemolysis can affect the interpretation of c-microRNAs signatures. They studied several oncological biomarkers reported in literature: many of them are highly expressed in blood cells. They demonstrated that this kind of c-microRNAs correlates with blood cell counts and that miR-122, which is not expressed in blood cells, doesn't follow this trend. Moreover, in hemolyzed plasma samples, red blood cell-associated microRNAs vary up to 30-fold compared to non-hemolyzed samples, further proving that c-microRNAs pool is affected by blood cells composition (Pritchard et al., 2012). For these reasons, sample handling and processing become extremely important. Duttagupta and colleagues tried to discriminate between whole blood microRNAs derived from blood cells—“contaminant microRNAs”—and what they called “truly circulating microRNAs”. Starting from whole blood samples and collecting different fractions from multiple centrifugation steps (Figure 3) they found that from fraction CS and S1 the content of “contaminant microRNAs” dropped, while the true c-microRNAs content stays more or less unchanged. On top of that, they showed that the variability of expression of marker c-microRNAs among a cohort of males and females decreases after the removal of the cellular contaminants originated from cellular microRNA signatures (Duttagupta et al., 2011). This points out how much the processing of the samples may affect the pool of c-microRNAs. Another study (Cheng et al., 2013) confirmed this variability, reporting that different plasma processing led, for the majority of c-microRNAs, to a variation in their expression levels, mainly due to different platelets and microvesicles content.

From the analytical point of view, it has to be considered that different extraction kits have distinct efficiencies in small RNAs recovery (Monleau et al., 2014). There are several challenges which involve also the detection and quantification of c-microRNAs. The design of qRT-PCR probes and assays is difficult because of (a) the shortness of microRNAs, (b) their wide range of concentrations, (c) the presence of many precursors and (d) the similarity in sequences. On the post-analytical side, we should mention that most of the detection methods rely on relative quantifications, therefore, an endogenous control is requested. This normalization analysis is needed in order to take into account the biological and technical inter-assay variability. So far, such control for c-microRNAs expression normalization, to be used for every tissue type, treatment and disease stage, has to be discovered yet. The most used in literature are miR-16, snRNA U6 and spiked-in cel-miR-39, but there is no general consensus from the scientific community and a different endogenous control is generally used for different purposes. For instance, among the several transcripts of U6, U6-1 was found to be have high variability and U6-2 was not detectable, in a study on sera from Hepatitis B infected patients and matched controls (Zhu et al., 2012). In two different plasma studies on CRC, miR-16 was found to have quite high stability and little variability between control and case patients (Ng et al., 2009; Huang et al., 2010). In another case, it has been reported, in serum samples from lung cancer patients, miR-16 being inconsistent, choosing to directly normalize the expression levels of target microRNAs to total RNA (Chen et al., 2008). Several authors have concluded that a universal endogenous control is unlikely to be discovered and a suitable reference should be assessed every time considering the different biological conditions of the samples. However, cost and sample requirements needed for the choice of several reference RNAs are not always possible, especially in a clinical or diagnostic setting.

## Conclusions

As described in the previous paragraphs, microRNAs have some advantages as high specificity, sensitivity, and classification power, which can be exploited for cancer personalized medicine. Furthermore, microRNAs are remarkably stable small molecules shown to be well preserved in FFPE as well as in fresh snap-frozen specimens and in biofluids.

microRNAs affect cancer biology being involved in all the hallmarks of cancer both as tumor suppressor and as onco-miR. They can be extracted from different biological sources and used as biomarkers in order to classify cancerous vs. non-cancerous tissues, distinguish different cancer types and also efficient cancer therapies. Thanks to the sensitive signatures, patterns of microRNAs may be able to follow the progression of cancer, being an important tool in clinical practice. In particular, detection of c-microRNAs, obtained by a non-invasive procedure, seems to be a new promising field with the potential of revolutionizing cancer diagnostics, increasing compliance of patients, ease of use and accessibility to these biomarkers. As a result of a growing use of high-throughput techniques, another emerging field in microRNA diagnostics is represented by SNPs analysis. miRSNP can affect microRNA expression and function, being present on microRNAs sequence, on their target genes or also in genes involved in their biogenesis. They can affect cancer susceptibility, prognosis and response to treatment.

In this review, we reported the main concepts on microRNA cancer personalized medicine. However, some issues have to be considered while going through the huge amount of studies on microRNA function and classification capacity.

Despite the advances obtained in the field, many open questions and challenges still remain to be addressed (Table 3). One limitation in microRNAs detection is represented by the closely similar sequences among microRNAs family members, their ancestral RNAs (pre-microRNAs and pri-microRNAs) and isomiRs (Thomas et al., 2010; Chugh and Dittmer, 2012; Zhou et al., 2012). Moreover, there are many methods which can be used to measure microRNAs including qRT-PCR, microarrays, next generation sequencing (NGS), and more recently, digital droplet PCR (ddPCR). These conventional methods have of course their own drawbacks, mainly time-consuming, expensive material and target modification steps for detection. Concerning c-microRNAs, the main limitations are represented by the small amount of RNA and the corresponding microRNAs extracted from biofluids. Indeed, RNA concentration is often under the detection limits of common spectrophotometric devices and this is the reason why for qRT-PCR, it is recommended to use a fixed volume rather than a fixed RNA amount. Furthermore, the right selection of a suitable normalization method to remove technical variations and increase the accuracy of microRNAs quantification is of enormous relevance. The issue of reference genes is especially critical in the quantification of c-microRNAs, due to the extremely low levels in biofluids of common reference genes (U6, miR-16, 5S rRNA, small nucleolar RNAs).

**Table 3:** Challenges on microRNA studies from basic microRNA analysis to microRNA functional studies.

microRNA analysis	**Source of preparation:** lack of standardized protocols
	**Discrimination between pri-, pre- and mature forms:** difficult to distinguish the different forms of microRNA maturation
	**Detection techniques:** lack of strong sensitivity and sensibility at single-base level
	**Short sequence for primer design**
	**Quantity in biofluids:** the low quantity of microRNAs in biofluids demands high-sensitivity techniques
	**Normalization methods:** the particular nature of microRNAs and their involvement in post-transcriptional regulation makes difficult to find an universal normalizer
microRNA functional studies	**High biological complexity:** their ability to target multiple mRNAs and the presence of multiple different target sites on a single mRNA creates a complex network of regulation difficult to untangle
	**Non-physiological conditions:** microRNAs generally act as fine-tuners of gene expression, thus, forced over-expression or inhibition in cellular or non-cellular systems are not representative of the reality
	**Few mechanicistic studies on miRSNP:** miRSNP have been studied mainly for their association to cancer risk but only few of these works try to unravel the mechanism underneath the shown effects

On the other hand, from a biological point of view, microRNAs biogenesis and function must be further explored in order to better understand mechanisms at the basis of their involvement in cancer. The commonly accepted mechanism of microRNA action and targeting involves the interaction between microRNA 5′-end (“seed region”) and mainly the mRNA 3′-UTR. However, target sites were found also in the coding sequence (CDS) and in 5′ UTR (Lytle et al., 2007; Kloosterman et al., 2016). It has been suggested that this preference could be due to the presence of ribosomes in CDS and translation initiation complexes in 5′ UTR which compete with the RISC complex (Bartel, 2004). Regarding the way of action, microRNAs contribute to gene expression regulation by fine-tuning rather than knocking-down their target mRNA. Thus, microRNAs over-expression performed for luciferase assay or western blot, the “gold standard” techniques to analyze their effect on targets, do not represent the real physiological situation. Although microRNAs exert slight effects on mRNAs, they adopt other strategies to potentiate their regulation. Indeed, one microRNA can simultaneously regulate multiple targets and different microRNAs can have a role as post-transcriptional regulators on the same target. In addition, microRNAs are often involved in feedback loops, thereby potentiating their suppression potential. A complex interplay exists also between transcriptional and post-transcriptional regulators (transcription factors and microRNAs) to orchestrate gene expression and signaling. Moreover, some microRNAs are able to regulate gene expression of their own biogenesis and processing factors, as Dicer (Ristori et al., 2015). Lastly, it is important to take into account the tissue specificity of microRNAs action, thus studies on single tissue-type are to be considered carefully. All these interactions and regulation levels lead to highly complex networks of microRNA/target pathways. For this reason, systems biology approach tries to obtain a more comprehensive understanding of miRNA regulatory structure, combining biological data acquisition and integration, network construction, mathematical modeling and experimental validation.

One important point to mention is that, recently, miRNA–target interaction knowledge has been enriched by the discovery of long non-coding RNAs (lncRNAs) (Yoon et al., 2014) and circular RNAs (circRNAs) (Hansen et al., 2013; Memczak et al., 2013) and circRNAs which could act as miRNA sponges, reducing their regulatory effect on mRNAs. One hypothesis is that all RNA transcripts containing binding sites for microRNAs can compete specifically for shared microRNAs, acting as competing endogenous RNAs (ceRNAs) (reviewed in Thomson and Dinger, 2016). This concept is extremely important not only for having a complete view of the mechanisms of action of the microRNAs, but also for their biomarker employment.

In the future new more accurate and PCR-free single base sensitive platform are needed. Therefore, a device in which sample-preparation steps (e.g., enzymatic steps for PCR-based amplifications) are removed, would represent an improvement in microRNAs detection and quantification. Some tentative approaches are under study like the integration of a dynamic chemistry for “Single Nucleobase Labeling” with a bead-based platform (Luminex®) (Venkateswaran et al., 2016), or the use of a power-free microfluidic chip involving a technology based on laminar flow–assisted dendritic amplification (LFDA) (Hasegawa et al., 2017). In the former case the same technology was used to detect microRNAs from serum samples (Rissin et al., 2017). On top of that, new methods to avoid normalization of signals are requested, like the use of ratio between the Ct of two different miRNAs (Sharova et al., 2016). As the detection techiques are fundamental, even the source of microRNAs affect the biological outcome. In this sense, exosomes are representing the future of biomarkers from liquid biopsies. More effort in studying this vehicles will help elucidate the mechanism for which microRNAs are realeased in biofluids, thus affecting our way to use them as biomarkers. In addition, the main step forward toward a safe and stable use in clinics of these tools will be the standardization of protocols regarding pre- and post analytical factors. Therefore, a standardized method for isolation of tissues of biofluids as well as preservation of the sample will drastically decrease variability of results, enhancing similarity and robustness of studies in literature. This point is still missing and it is of critical relevance.

In conclusion, microRNAs are fundamental regulator of cell life, linking all its biological functions. Although their analysis has some challenges, the above-mentioned advantages reveal microRNAs as important tools for biomarkers investigation. If this field will be further pursued, clinicians could be guided by simple tests detecting pattern of microRNAs expression or even single nucleotide variations, making them strongly valid for cancer personalized medicine.

## Author contributions

SD designed the work thinking how it should have been organized, drafted and wrote the manuscript, draw the figures and revised the manuscript critically. MG contributed to the conception and design of the work, supporting the revision for important intellectual content. VDV revised the work critically. MD gave overall intellectual critical support and approved the version to be published. All authors read and approved the final manuscript.

### Conflict of interest statement

The authors declare that the research was conducted in the absence of any commercial or financial relationships that could be construed as a potential conflict of interest.
